# Clinical characteristics of necrotizing enterocolitis occurring within the first week of life in low-birth-weight preterm infants: a multicenter retrospective study

**DOI:** 10.3389/fmed.2026.1940394

**Published:** 2026-09-09

**Authors:** Jiexia Gao, Jie Fu, Fan He, Yanbin An, Jinglin Xu, Bowen Weng, Chongbing Yan, Yuanyang Zhang, Na Zhuo, Jiahuai Liu, Cong Yu, Dongmei Chen, Arigonggaowa, Cheng Cai

**Affiliations:** 1 Department of Neonatology, Shanghai Children’s Hospital, School of Medicine, Shanghai Jiao Tong University, Shanghai, China; 2 Department of Neonatology, Bozhou Children’s Hospital, The People’s Hospital of Bozhou, Bozhou, Anhui, China; 3 Neonatal Intensive Care Unit, Jiangxi Provincial Children’s Hospital, Nanchang, Jiangxi, China; 4 Neonatology Division, Inner Mongolia Maternity and Child Health Care Hospital, Hohhot, Inner Mongolia, China; 5 Department of Neonatology, Quanzhou Maternity and Children’s Hospital, Quanzhou, Fujian, China

**Keywords:** clinical characteristics, early onset, low birth weight, necrotizing enterocolitis, preterm infants

## Abstract

**Objectives:**

This study aimed to compare the perinatal background, clinical characteristics, and outcomes of low-birth-weight preterm infants with Necrotizing enterocolitis (NEC) occurring within the first week of life and those with NEC occurring after the first week of life.

**Methods:**

This multicenter retrospective study included preterm infants with a birth weight <2,500 g who were diagnosed with NEC within 28 days of life across five neonatal intensive care units from January 2021 to June 2024. Infants were classified according to age at NEC onset into an early-onset NEC group (EO-NEC, ≤7 days) and a later-onset NEC group (LO-NEC, >7 days). Perinatal characteristics, disease severity, hematological parameters, imaging findings, treatment, and short-term outcomes were compared between groups.

**Results:**

Among 405 eligible infants, 151 (37.3%) developed EO-NEC. Compared with LO-NEC infants, EO-NEC infants had higher gestational age and birth weight but were more likely to be small for gestational age (SGA) and exposed to maternal hypertensive disorders. EO-NEC was also associated with a higher proportion of non-full enteral feeding before onset, Bell stage III disease, intestinal perforation, thrombocytopenia, and higher hemoglobin and hematocrit levels. However, surgical intervention and mortality did not differ significantly between groups.

**Conclusion:**

These findings suggest that NEC occurring within the first week of life has several characteristic clinical features and may help identify infants who require closer abdominal and hematological monitoring.

## 1 Introduction

NEC is a severe and potentially life-threatening gastrointestinal disease that primarily affects preterm and low-birth-weight infants (1). NEC typically occurs during the first few weeks after birth and is characterized by nonspecific clinical manifestations, including feeding intolerance, abdominal distension, vomiting and bloody stools. These symptoms may overlap with other neonatal conditions, such as sepsis, making early diagnosis challenging. In severe cases, NEC can progress rapidly to intestinal necrosis, perforation, diffuse peritonitis, systemic infection, shock, and death. Although the pathogenesis of NEC remains incompletely understood, intestinal dysbiosis, immature immune regulation, impaired intestinal barrier function, and inflammatory injury are considered important contributors to disease development (2, 3).

The timing of NEC onset varies among preterm infants and may be influenced by gestational age and birth weight. Previous cohort data showed that, among infants <33 weeks' gestation, NEC appeared to present at a mean age of approximately 7 days in relatively more mature infants, whereas onset was delayed to approximately 32 days in smaller infants with lower gestational age (4, 5). These findings suggest that NEC onset timing may reflect differences in infant maturity and perinatal background. However, the clinical relevance of NEC occurring within the first week of life remains unclear, particularly among low-birth-weight preterm infants. Although a few studies have compared early- and late-onset NEC in term infants and reported that early-onset NEC may be associated with higher gestational age and more severe clinical manifestations, comparable data in preterm infants with low birth weight are limited (6).

In clinical practice, hematological tests and abdominal imaging are commonly used to assess infants with suspected NEC. Blood cell parameters, including platelet count, white blood cell count, and neutrophil count, have been increasingly investigated as potential indicators of NEC severity, surgical intervention, and mortality (7). However, whether these routinely available clinical and laboratory findings differ according to NEC onset timing has not been well characterized in low-birth-weight preterm infants.

Therefore, this study aimed to characterize NEC occurring within the first week of life in low-birth-weight preterm infants by comparing it with NEC occurring after the first week of life.

## 2 Methods

### 2.1 Ethics statement

This was a multicenter retrospective cohort study conducted at five pediatric hospitals in China: Shanghai Children's Hospital, Jiangxi Children's Hospital, Inner Mongolia Maternal and Child Health Hospital, Fujian Quanzhou Children's Hospital, and Bozhou Children's Hospital. Medical records of neonates diagnosed with NEC between January 2021, and June 2024 were reviewed. The study protocol was approved by the Ethics Committee of Shanghai Children's Hospital (Approval No. 2026R094-E01). The study was conducted in accordance with the Declaration of Helsinki.

### 2.2 Data collection

This study included preterm infants with low birth weight (<2,500 g) who were diagnosed with NEC within 28 days of life. Infants were classified by postnatal age at NEC onset into an early-onset NEC group (EO-NEC, ≤7 days) and a later-onset NEC group (LO-NEC, >7 days). The 7-day cutoff was chosen based on previous cohort findings and was used to compare clinical features between infants with NEC occurring within and after the first week of life.

Demographic, perinatal, clinical, laboratory, imaging, treatment, and outcome data were retrospectively collected from medical records. These included sex, gestational age, birth weight, 5 min Apgar score, delivery mode, maternal complications, antenatal steroid exposure, placental abnormalities, age at NEC onset, Bell stage, feeding status, feeding type, intestinal perforation, surgical intervention, length of hospital stay, and mortality. Bell stage III disease was analyzed as an indicator of advanced NEC. Non-full enteral feeding was defined as failure to achieve full enteral nutrition before NEC onset. Laboratory parameters within 3 days before onset included white blood cell count, absolute neutrophil count, platelet count, hemoglobin, and hematocrit. Thrombocytopenia was defined as a platelet count <150 × 10^9^/L. Cranial ultrasound findings before onset were also recorded.

### 2.3 Statistical analysis

Statistical analyses were performed using R Studio software and SPSS Statistics. Continuous variables with a normal distribution were expressed as mean ± standard deviation (SD) and compared using the independent Student's *t*-test. Non-normally distributed variables were presented as median and interquartile range [M (P25–P75)] and compared using the Mann–Whitney *U* test. Categorical variables were expressed as counts and percentages [*n* (%)] and compared using the chi-square test or Fisher's exact test. Variables with *P* < 0.10 in the univariable analysis were considered candidate variables for an exploratory multivariable logistic regression analysis to evaluate clinical factors associated with NEC occurring within the first week of life. A two-sided *P* < 0.05 was considered statistically significant.

**Figure 1:**
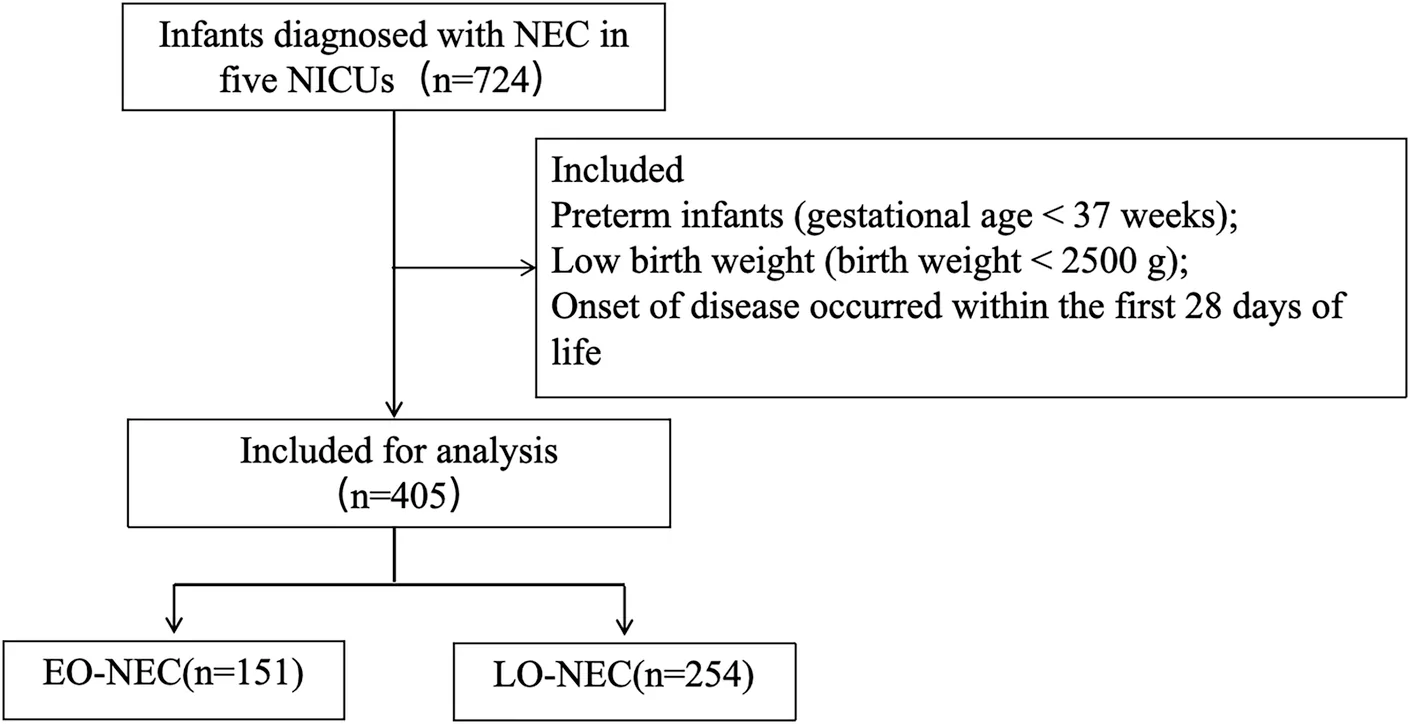
Flow chart of infant selection and grouping.

Figure 1 shows the overall workflow of the study.

## 3 Results

A total of 724 infants diagnosed with NEC were identified across the participating centers. Among them, 405 infants met the inclusion criteria and were included in the final analysis. Of these, 151 infants (37.3%) developed NEC within the first week of life and were assigned to the EO-NEC group, while 254 infants (62.7%) developed NEC after the first week of life and were assigned to the LO-NEC group. The median age at NEC onset for the overall cohort was 11 days.

### 3.1 Baseline information

Baseline and perinatal characteristics are summarized in Table 1. Compared with the LO-NEC group, infants in the EO-NEC group had significantly higher gestational age and birth weight (*P* < 0.05). The proportion of small for gestational age (SGA) infants was also significantly higher in the EO-NEC group (*P* < 0.05). The 5 min Apgar score was slightly higher in the EO-NEC group (*P* < 0.05).

**Table 1:** Perinatal and baseline characteristics of infants in EO-NEC and LO-NEC groups.

Variable	Total (*n* = 405)	Early onset (*n* = 151)	Late onset (*n* = 254)	*χ* ^2^ */z*	*P*
	*n*%(*n*), M(P_25_-P_75_)				
Male	59.8 (242)	62.9 (95)	57.8 (147)	1.000^a^	0.317
Gestational age, week	32.0 (30.1, 34.0)	33.5 (31.1, 35.0)	31.4 (29.9, 33.4)	−5.740^b^	<0.001
Birth weight, g	1,595 (1,270, 2,000)	1,780 (1,390, 2,140)	1,500 (1,240, 1,850)	−4.151^b^	<0.001
5-min Apgar score	9 (9, 10)	9 (9, 10)	9 (9, 10)	−2.850^b^	0.004
SGA	21.0 (84)	33.1 (49)	13.9 (35)	22.035^a^	<0.001
Invasive respiratory support	12.6 (51)	8.0 (12)	15.4 (39)	7.695^a^	0.053
Vaginal delivery	33.1 (134)	28.5 (43)	35.8 (91)	2.311^a^	0.128
Hypertensive disorder in pregnancy	23.0 (93)	29.1 (44)	19.2 (49)	5.192^a^	0.023
IAI	13.1 (53)	13.9 (21)	13.1 (32)	0.143^a^	0.706
ICP	2.7 (11)	3.3 (5)	2.4 (6)	0.323^a^	0.570
PROM	27.7 (112)	16.5 (25)	34.2 (87)	14.822^a^	<0.001
Antenatal steroid	47.9 (187)	51.3 (76)	45.8 (111)	1.106^a^	0.293
Placental abnormalities				0.443^a^	0.801
Placental abruption	45	15	30		
Placenta previa	12	4	8		
None	348	132	216		

SGA, small for gestational age; IAI, intra-amniotic inflammation; ICP, intrahepatic cholestasis of pregnancy; PROM, prolonged rupture of the membranes.

a Chi-square test.

b Mann–Whitney *U* test.

Regarding maternal and perinatal factors, hypertensive disorders during pregnancy were more common in the EO-NEC group (*P* < 0.05), while PROM was significantly more prevalent in the LO-NEC group (*P* < 0.05). There were no significant differences between groups in terms of sex, delivery mode, intra-amniotic inflammation (IAI), intrahepatic cholestasis of pregnancy (ICP), use of antenatal steroids, or need for invasive respiratory support. Additionally, there was no significant difference in the incidence of placental abruption or placenta previa between the two groups.

### 3.2 Clinical features

As shown in Table 2, the EO-NEC group had a significantly higher proportion of Bell stage III disease than the LO-NEC group (*P* < 0.05), whereas the rate of surgical intervention did not differ significantly between groups.

**Table 2:** Clinical features in EO-NEC and LO-NEC groups.

Variable, %(*n*), *n*	Early onset (*n* = 151)	Late onset (*n* = 254)	*χ* ^2^ */z*	*P*
NEC severity category			4.015^a^	0.045
Stage III	25.2 (38)	16.9 (43)		
Surgical intervention	26.5 (40)	23.6 (60)	0.419^a^	0.517
Non-full enteral feeding before onset	80.1 (121)	61.4 (156)	20.399^a^	<0.001
Feeding before onset			5.739^a^	0.220
Exclusive breast milk	27	56		
Formula feeding	77	132		
Mixed feeding	28	48		
Blood transfusion within 1 week before onset	7.3 (11)	12.6 (32)	3.371^a^	0.185
Intestinal perforation	14.6 (22)	8.2 (21)	3.963^a^	0.047
PDA before onset	29.1 (44)	37.0 (94)	2.610^a^	0.106
Shock before onset	10.6 (16)	13.8 (35)	0.872^a^	0.350
Sepsis before onset	31.2 (47)	38.6 (98)	2.291^a^	0.130
Length of hospital stay	29 (17, 45)	39 (25, 56)	−4.126^b^	<0.001
Mortality	6.8 (10)	4.8 (12)	2.515^a^	0.473

PDA, patent ductus arteriosus.

a Chi-square test.

b Mann–Whitney *U* test.

A significantly higher proportion of infants in the EO-NEC group had not achieved full enteral feeding before NEC onset compared with those in the LO-NEC group (80.1% vs. 61.4%). However, the distribution of feeding type before onset, including exclusive breast milk, formula feeding, and mixed feeding, was not significantly different between groups.

Among clinical factors, intestinal perforation was significantly more frequent in the EO-NEC group (*P* < 0.05). Given the differences in gestational age and birth weight between the two groups, we further adjusted for these two factors. EO-NEC remained significantly associated with Bell stage III disease and intestinal perforation after adjustment (Supplementary Table 3). No significant differences were observed between groups in blood transfusion within one week before onset, patent ductus arteriosus, shock, or sepsis before onset. The LO-NEC group had a significantly longer median hospital stay than the EO-NEC group, while mortality did not differ significantly between groups.

### 3.3 Hematological and neuroimaging findings

As shown in Table 3, infants in the EO-NEC group had significantly lower platelet counts than those in the LO-NEC group (217.08 ± 107.35 vs. 313.47 ± 142.61 × 10^9^/L, *P* < 0.05). The frequency of thrombocytopenia was also higher in the EO-NEC group. The difference in platelet count remained significant after adjustment for gestational age and birth weight (Supplementary Table 3). In contrast, hemoglobin levels and hematocrit values were significantly higher in the EO-NEC group than in the LO-NEC group. White blood cell count and absolute neutrophil count were slightly lower in the EO-NEC group, but the differences were not statistically significant.

**Table 3:** Hematologic and imaging differences between EO-NEC and LO-NEC groups.

Variable, %(*n*), Mean ± S.D	Early onset (*n* = 151)	Late onset (*n* = 254)	*χ* ^2^ */t*	*P*
White blood cell count, ×10^9^/L	10.13 ± 6.32	11.43 ± 6.67	−1.929^b^	0.054
Absolute neutrophil count, ×10^9^/L	5.69 ± 3.85	6.24 ± 5.34	−0.090^b^	0.928
Platelet count, ×10^9^/L	217.08 ± 107.35	313.47 ± 142.61	−7.685^b^	<0.001
Thrombocytopenia	29.1 (44)	13.4 (34)	15.113^a^	<0.001
Hemoglobin, g/L	151.05 ± 33.83	128.02 ± 26.94	7.119^b^	<0.001
Hematocrit, %	44.59 ± 9.42	37.41 ± 7.70	7.904^b^	<0.001
Intraventricular hemorrhage before onset	18.5 (20)	23.7 (46)	9.162^a^	0.010
Periventricular leukomalacia before onset	0.6 (1)	0.3 (1)	1.562^a^	0.458
Periventricular white matter echogenicity	1.2 (2)	7.4 (19)	7.448^a^	0.024
Ventricular dilatation before onset	7.9 (12)	6.3 (16)	1.484^a^	0.476

a Chi-square test.

b *t*-test.

Regarding cranial ultrasound findings before NEC onset, the EO-NEC group had lower rates of intraventricular hemorrhage and periventricular white matter echogenicity than the LO-NEC group (P < 0.05). No significant differences were observed in periventricular leukomalacia or ventricular dilatation between groups.

### 3.4 Comparison of clinical characteristics between surgical and conservative treatment groups in infants with EO-NEC

In the subgroup of infants with EO-NEC, a comparison of clinical characteristics between the conservative treatment group and the surgical intervention group is shown in Supplementary Table 1. Infants in the surgical group had significantly lower birth weight than those in the conservative group (*P* < 0.05). The incidences of IAI, PDA before onset, sepsis before onset, and shock before onset were all significantly higher in the surgical group (*P* < 0.05). Intestinal perforation was markedly more frequent in the surgical group (*P* < 0.05). Thrombocytopenia was also significantly more common in the surgical group compared with the conservative group (*P* < 0.05). There were no significant differences between the two groups in gestational age, 5-min Apgar score, or age of onset (*P* > 0.05). In addition, the incidence of BPD after NEC onset was significantly higher in the surgical group than in the conservative group (*P* < 0.05).

### 3.5 Factors associated with EO-NEC

Multivariable logistic regression was performed to evaluate factors associated with NEC occurring within the first week of life. Platelet count (OR = 0.995, 95% CI: 0.993–0.997, *P* < 0.001), SGA status (OR = 0.377, 95% CI: 0.221–0.644, *P* < 0.001), and non-full enteral feeding before onset (OR = 0.476, 95% CI: 0.269–0.843, *P* *=* 0.011) were independently associated with EO-NEC (Supplementary Table 2).

## 4 Discussion

In this multicenter cohort of low-birth-weight preterm infants with NEC, infants who developed NEC within the first week of life showed distinct clinical characteristics compared with those who developed NEC later. EO-NEC infants had higher gestational age and birth weight but were more likely to be SGA and exposed to maternal hypertensive disorders during pregnancy. They also showed higher proportions of Bell stage III disease and intestinal perforation, lower platelet counts, a higher frequency of thrombocytopenia, and higher hemoglobin and hematocrit levels. However, surgical intervention and mortality were not significantly different between groups.

The 7-day cutoff was chosen based on previous cohort findings showing that NEC may occur around the first week of life in relatively more mature preterm infants (6). In our cohort, infants with EO-NEC also had higher gestational age and birth weight than those with LO-NEC, supporting the observation that NEC onset timing may be related to infant maturity.

A notable finding of this study was that EO-NEC infants were relatively more mature but had a higher proportion of SGA status. SGA reflects impaired fetal growth and is often associated with chronic intrauterine hypoxia and reduced intestinal perfusion, which may increase intestinal vulnerability shortly after birth (8). The higher frequency of maternal hypertensive disorders in the EO-NEC group further supports the possible role of placental insufficiency and impaired fetal blood flow. Under these conditions, redistribution of fetal circulation may preserve blood supply to vital organs while reducing gastrointestinal perfusion, thereby contributing to earlier intestinal injury and NEC presentation (9–11).

More infants in the EO-NEC group had not reached full enteral feeding before NEC onset, suggesting that NEC within the first week of life often occurs during the early stage of feeding establishment. Feeding practice is closely related to NEC prevention, including the timing of feed initiation, feeding advancement, and human milk use. However, previous studies have not shown clear evidence that slower feeding advancement reduces NEC or death in very preterm or very-low-birth-weight infants, and slower advancement may delay full enteral feeding and increase infection risk (12–16). Further studies are needed to clarify the relationship between feeding practices and NEC onset timing.

EO-NEC infants had higher proportions of Bell stage III disease and intestinal perforation, whereas surgical intervention and mortality were similar between groups. This suggests that NEC occurring within the first week of life may have more prominent severe-stage and perforation-related features. However, treatment decisions and survival are likely influenced by multiple factors, including overall maturity, systemic condition, timing of diagnosis, and center-specific management strategies. The longer hospital stay in the LO-NEC group may be partly related to their lower gestational age, and lower birth weight (17, 18).

In recent years, an increasing number of studies have identified hematological parameters such as WBC, PLT, ANC as potential risk factors for NEC severity and surgical intervention (13, 19, 20). Huang et al. suggested that thrombocytopenia and hypoalbuminemia may be independent predictors of intestinal perforation in neonates with NEC (21). Li et al. found that a platelet count less than 100 × 10^9^/L and a lymphocyte count less than 1.5 × 10^9^/L were independent risk factors for rapidly progressive NEC (22). It has been reported that thrombocytopenia, defined as a platelet count below 150 × 10^9^/L, occurs in 50 to 95 percent of NEC infants during the clinical course (23).

In our study, the EO-NEC group had significantly lower platelet counts compared to the LO-NEC group. This suggests that thrombocytopenia may be an important hematological feature of NEC occurring within the first week of life. The current explanation suggests that decreased platelet levels could lead to microcirculatory dysfunction and thrombosis, which contribute to activate endothelial cells and macrophages and the release of inflammatory cytokines and nitric oxide (24, 25). These processes may trigger a systemic inflammatory response and contribute to the progression of NEC.

In addition, the EO-NEC group had lower rates of intraventricular hemorrhage and periventricular white matter echogenicity but also had a significantly higher gestational age. These differences may partly relate to the difference in gestational age. As our study did not include long-term neurodevelopmental follow-up, future studies are needed to further evaluate the neurodevelopmental outcomes of infants with EO-NEC.

We also performed multivariable analysis to assess factors associated with EO-NEC. Platelet count, SGA status, and non-full enteral feeding before onset were associated with EO-NEC. In the EO-NEC subgroup, infants who underwent surgery had lower birth weight and higher rates of intra-amniotic inflammation, PDA before onset, sepsis, shock, intestinal perforation, thrombocytopenia, and BPD after onset than those managed without surgery. These findings suggest that EO-NEC infants requiring surgery had more severe clinical conditions. The higher rate of thrombocytopenia in these infants also supports close abdominal and hematological monitoring during the early stage of disease.

This study has several strengths. It included data from five neonatal intensive care units and focused on NEC onset timing in low-birth-weight preterm infants, a population with limited available data. By comparing NEC occurring within and after the first week of life, this study showed that early-onset cases had different perinatal, clinical, and hematological features. This study also has limitations. Its retrospective design may have introduced selection bias and residual confounding. Inter-center differences in feeding practices, clinical management, and data collection may have affected the findings. Further prospective studies are needed to confirm these results.

## 5 Conclusion

In this multicenter retrospective study, NEC occurring within the first week of life in low-birth-weight preterm infants had several characteristic clinical features, including higher gestational age and birth weight, more frequent SGA status and maternal hypertensive disorders, lower platelet counts, and higher rates of Bell stage III disease and intestinal perforation. These findings may help guide abdominal and hematological monitoring during the early postnatal period.

## Data Availability

The original contributions presented in the study are included in the article/Supplementary Material, further inquiries can be directed to the corresponding authors.
