# Supplementary material for: Clinical characteristics of necrotizing enterocolitis occurring within the first week of life in low-birth-weight preterm infants: a multicenter retrospective study
Source: Front Med (Lausanne). 2026 Sep 9;13:1940394. doi: 10.3389/fmed.2026.1940394 (PMC13597887; doi:10.3389/fmed.2026.1940394)
Supplement: Supplementary file 1 [file Table1.docx]

**Supplementary**

Table 1 Comparison of clinical characteristics between surgical and conservative treatment groups in infants with EO-NEC

| Variable, %(n), Mean±S.D, M(P_25_-P_75_) | Conservative (n=111） | Surgical (n=40) | *χ*^2^*/z/t* | *P* |
| --- | --- | --- | --- | --- |
| Gestational age, week | 33.7（31.4，35.3） | 32.9（29.9，34.5） | -1.561^b^ | 0.119 |
| Birth weight, g | 1830（1460，2200） | 1585（1200，1970） | -2.369^b^ | 0.018 |
| 5-minute Apgar score | 9（9，10） | 9（9，10） | -0.406^b^ | 0.684 |
| Age of onset | 4.08±2.08 | 4.15±1.92 | -0.198^c^ | 0.843 |
| IAI | 8.1(9) | 30(12) | 11.770^a^ | <0.001 |
| PDA before onset | 22.5(25) | 47.5(29) | 8.884^a^ | 0.003 |
| Sepsis before onset | 21.6(24) | 57.5(23) | 17.656^a^ | <0.001 |
| Shock before onset | 5.4(6) | 25(10) | 11.917^a^ | <0.001 |
| Intestinal perforation | 1.8(2) | 50(40) | 54.879^a^ | <0.001 |
| Thrombocytopenia | 21.6（24） | 50（20） | 11.468^a^ | <0.001 |
| BPD | 10.8（12) | 42.5(17) | 19.029^a^ | <0.001 |
| Length of hospital stay | 32.5(21,62) | 27.5(16, 39) | -4.126^b^ | <0.001 |

BPD, bronchopulmonary dysplasia; *a:chi-square test, b: Mann-Whitney U, c: t-test*

Table 2 Multivariable logistic regression analysis of factors associated with EO-NEC

| Variable | *β* | *SE* | *Wald χ*^2^ | *P* | *OR* | 95%*CI* |
| --- | --- | --- | --- | --- | --- | --- |
| Platelet Count | -0.005 | 0.001 | 22.299 | <0.001 | 0.995 | 0.993-0.997 |
| Non-Full enteral feeding before onset | -0.742 | 0.291 | 6.479 | 0.011 | 0.476 | 0.269-0.843 |
| SGA | -0.975 | 0.273 | 12.784 | <0.001 | 0.377 | 0.221-0.644 |

Table 3 Adjusted analyses of selected clinical characteristics between EO-NEC and LO-NEC

| Variable | Effect estimate | 95%*CI* | *P* |
| --- | --- | --- | --- |
| Bell stage III NEC | aOR = 2.362 | 1.382–4.034 | 0.002 |
| Intestinal perforation | aOR = 3.054 | 1.528–6.107 | 0.002 |
| Platelet count | Adjusted mean difference = −113.699×10⁹/L | −139.547 to −87.850 | <0.001 |
